# Tuning the electrochemical performance of a copper-based 2D rectangular layered metal organic framework by incorporating reduced graphene oxide and polyaniline

**DOI:** 10.1039/d5ra05415j

**Published:** 2026-03-17

**Authors:** Muhammad Shahbaz, Madiha Riasat, Ghulam Ullah, Muhammad Waheed Mushtaq, Maham Saeed, Sundas Shahzad, Ayesha Shahzad, Zeeshan Mustafa, Onur Şahin, Shahzad Sharif

**Affiliations:** a Materials Chemistry Laboratory, Institute of Chemical Sciences, Government College University Lahore 54000 Pakistan mssharif@gcu.edu.pk +92 345 4579334; b Department of Chemistry, Govt. Graduate College of Science Wahdat Road Lahore Pakistan; c University of Chinese Academy of Sciences Beijing 101408 PR China; d Department of Occupational Health & Safety, Faculty of Health Sciences, Sinop University TR-57000 Sinop Turkey

## Abstract

To meet the challenge of developing efficient energy storage devices, metal organic frameworks (MOFs) with intrinsic properties have emerged as promising candidates. The conductivity and stability of the extended 1D π–*d* and π–π stacking in a 2D MOF can be further enhanced by fabricating its composite with conductive materials. Herein, a nitrogen-containing 2D Cu-PDA MOF was synthesized and mixed with conductive materials, such as reduced graphene oxide (rGO) and polyaniline (PANI), to enhance the electrical conductivity. After structural investigation, the electrochemical attributes of Cu-PDA and its composites (Cu-PDA@rGO and Cu-PDA@PANI) have been explored by utilizing different electroanalytical techniques like CV, GCD and EIS analyses. In a three-electrode assembly, Cu-PDA@rGO shows a specific capacity of 551.31 C g^−1^. Hence, for practical applications, a hybrid supercapacitor has been designed by fabricating Cu-PDA@rGO against activated carbon (AC), which reveals a specific capacity of 159.4 C g^−1^, a specific energy of 32.10 Wh kg^−1^ and a specific power of 180.17 W kg^−1^ while maintaining a coulombic efficiency of 99.4% even after 5000 GCD cycles. These excellent findings demonstrate that Cu-PDA@rGO is a potential candidate for future energy storage devices.

## Abstract 2

The conductivity and stability of the extended 1D π–*d* and π–π stacking of a 2D MOF can be further enhanced by fabricating its composites with conductive materials.

## Graphical abstract

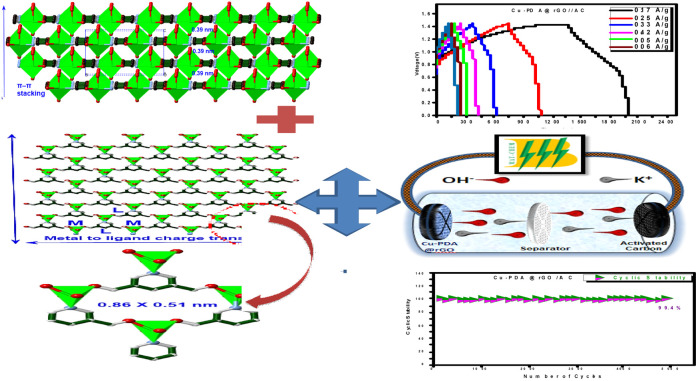

## 1 Introduction

Energy technology is essential for driving societal progress.^1,2^ There is a pressing requirement to develop a low-cost energy storage solution using renewable resources to mitigate the ongoing large-scale reliance on fossil fuels for energy generation.^3,4^ Traditionally, batteries and supercapacitors are the predominant energy storage systems.^5,6^ Batteries derive energy from the charge transfer reactions occurring at their electrode surfaces and are characterized by high specific energies.^7,8^ However, their limitations include the inability to deliver high power, and their normal functioning is hindered when high power extraction is required.^9,10^ Conversely, electrochemical capacitors or supercapacitors store charge at the electrode–electrolyte interface through the development of electric double layer capacitors (EDLCs).^11^ The advantages of supercapacitors include their contribution to green energy technology, high power output, rapid charge–discharge rate and long life stability.^12,13^ In extreme circumstances, such as power outages, supercapacitors serve as backup power sources to complement other energy storage devices, like batteries and fuel cells.^14,15^ However, they are limited by their inability to provide the high specific energy that a battery system can achieve, while they also generate a substantial capacitance at elevated operating voltages.^16^ The inability of both battery and supercapacitor technologies to deliver high energy and specific power simultaneously has motivated researchers to explore alternative energy storage solutions. In this field, a synergistic method for combining the characteristics of both batteries and supercapacitors into a new device presents an attractive research opportunity. This innovative energy technology is recognized as a supercapattery or hybrid supercapacitor.^17,18^ This device is fundamentally based on the integration of two types of electrode materials: battery-grade cathodes and capacitive-grade anodes.^19,20^ This configuration allows the extraction of high specific energy from the battery-grade cathode, while the capacitive-grade anode provides high specific power and longer cyclic stability.^21,22^

Metal organic frameworks (MOFs) have high surface area, rich porosity,^23^ stability, crystallinity^24^ and tunable morphology.^25–27^ However, a notable disadvantage of MOFs is their low conductivity.^28,29^ To address this issue, researchers have been exploring various strategies including the use of ligands with conjugation^30,31^ and mixing conductive materials with MOFs.^32,33^ Recently, the Cu-MOF derived from *para*-phthalic acid (PTA) was fabricated in an asymmetric device that achieved specific capacity, specific energy and specific power values of 152.2 C g^−1^, 35.9 Wh kg^−1^ and 5950 W kg^−1^, respectively, with 92.15% of stability after 5000 GCD cycles.^34^ In contrast, a bi-linker copper-based MOF originating from pyromellitic acid and 2-methylimidazole was also reported, which demonstrated an improved performance, achieving specific capacity, specific energy, and specific power of 230 C g^−1^, 52 Wh kg^−1^ and 4000 W kg^−1^, respectively, with 93% cyclic stability after 5000 GCD cycles.^35^ It is evident that the presence of a heteroatom improves the electrochemical performance of MOFs. In addition to tuning the structure of linkers, the incorporation of conductive materials, such as graphene and polyaniline, has become a prime focus of researchers. *In situ* polymerized graphene oxide (GO) was reported with enhanced thermal stability of wood-based composites.^36^ Furthermore, the electrochemical anodic oxidation technique was utilized to fabricate a bamboo-like SiC nanowire array.^37^ Zhao *et al.* engineered a NiCo_2_S_4_/rGO composite through the sequential addition of a cobalt-based framework to graphene oxide, followed by etching, hydrolysis, introduction of Ni^2+^ and high temperature vulcanization. This composite achieved a specific capacitance of 272.5 mAh g^−1^ at a current density of 1 A g^−1^, while the assembled asymmetric device showcased a specific energy of 59.6 Wh kg^−1^ with 799 W kg^−1^ specific power.^38^ Similarly, Wu *et al.* synthesized a Co-Ni/rGO hybrid composite derived from MOFs. An asymmetric device was designed using an rGO aerogel anode, resulting in a specific energy of 39.58 Wh kg^−1^ and specific power of 208.3 W kg^−1^ with a remarkable capacity retention of 87.4% after 10 000 GCD cycles.^39^ Sm-MOF/rGO/PANI composites were also reported to have high surface tunability, which was utilized as electrode components of supercapacitor devices, achieving a high specific capacitance of 218 F g^−1^ with a specific energy and specific power of 59.3 Wh kg^−1^ and 581 W kg^−1^, respectively.^40^ Meanwhile, a binder-free engineering strategy was reported to create Ni_3_(BTC)@PANI-rGO nanocomposites with a high energy, specific power, and specific capacity of 73.99 Wh kg^−1^, 848.29 W kg^−1^, and 1680 C g^−1^ respectively.^41^ MOF-199/PANI composite demonstrated a superior electrochemical performance, showing a specific capacity and specific energy of 272 C g^−1^ and 64 Wh kg^−1^, with maintaining cyclic stability of the 92% over 1000 GCD cycles, respectively.^42^ An rGO/Zr-MOF@PANI composite was also developed using a synergistic approach, which exhibited a specific capacitance of 1560.3 F g^−1^ and an impressive capacity retention rate of 92.7%.^43^ The electrochemical performance of ZIF-67 was also stretched using GO and NiS, which showed a specific energy of 88.2 Wh kg^−1^ at a current density of 1 A g^−1^.^44^ Biporous Cu-MOF/rGO composites also exhibited a mixed faradaic and non-faradaic electrochemical behavior with a maximum specific capacity and specific energy of 366.6 F g^−1^ and 14.66 W h kg^−1^, respectively.^45^ Composites of MOF with PANI and rGO have also been reported, which have an energy density of 25.11 Wh kg^−1^ and a power density of 860 W kg^−1^.^46^ The literature survey shows that making a composite of MOF improves the electrochemical performance, but not much attention has been made on the use of ligands having heteroatom (N,O,S) with lone pairs that facilitate electron transfer.^47,48^

In this study, a pyridine-3,5-dicarboxylic acid ligand was used to synthesize Cu-PDA-MOF. In order to exploit its full potential, the synthesized material was combined with reduced graphene oxide (rGO) and polyaniline (PANI) in a 1 : 1 ratio, yielding Cu-PDA@rGO and Cu-PDA@PANI composites. Electrochemical properties were elucidated using different electroanalytical techniques, like cyclic voltammetry (CV), galvanostatic charge–discharge (GCD) and electrochemical impedance spectroscopy (EIS). A three-electrode set-up demonstrated that Cu-PDA@rGO had outstanding charge storage capabilities. Hence, an asymmetric supercapacitor device consisting of Cu-PDA@rGO as the positive electrode and activated carbon (AC) as the negative electrode was fabricated. This hybrid device demonstrated remarkable electrochemical performance and impressive Coulombic efficiency. Dunn's method was also applied to evaluate the percentage contributions of capacitive and diffusive behaviors in the device. Various copper-based MOF materials have been reported, but they did not exhibit appreciable specific capacity. The novelty of this study revolves around the introduction of a heteroatom linker containing a nitrogen atom along with a carboxylic group, the rational choice of conductive agents, composition and structure. Pyridine-3,5-dicarboxylic acid generated a Cu–N environment in the framework, yielding a redox active and stable Cu-MOF, which is rarely reported. A combination of highly accessible electroactive sites in Cu-PDA-MOF with highly conductive rGO sheets provided improved interfacial interactions and better mechanical stability. Hence, superior charge transportation and electric conductivity were achieved in comparison to previously reported Cu-MOF@Carbon composites. These excellent findings prove that Cu-PDA@rGO is a potential contender for future energy storage to meet the demands of contemporary energy technology. Table 1 shows the comparison of the present study with reported materials, and there exist several MOF-based supercapacitors.^49–52^ The practical implications of this study revolve around two aspects. First, the Coulombic efficiency (99.4%) of Cu-PDA@rGO suggests that the device is long-term stable and highly durable in an aqueous medium, which is crucial for energy storage devices meant to be used at the grid scale. Second, the use of polyamine-based coordination polymers aligns with sustainable and environmentally benign electrode materials. This study can bridge the gap between water stable MOFs and practical applications at the industrial level.

**Table 1:** Comparison of the present work with reported studies on materials in two-electrode systems

Sample	CV scan rate (mV s^−1^)	Electrolyte	PW(V)	Specific capacity (C g^−1^)	*E*_s_/*P*_s_ (Wh kg^−1^)/W kg^−1^	CS/CE	Ref
rGO/Ppy/Zn-MOF	5–500	6 M KOH	0–1.8	175	19.68/1792	82/7000	53
Co-MOF/PANI	5–100	1 M KOH	0–1.6	104	23.2/1600	100/3000	54
Cu:NiO@rGO:PANI/NF	10–300	3 M KOH	0–1.4	81.9	25.6/1297.6	97.6/10 000	55
rGO/PANI composite	5–100	1 M H_2_SO_4_	0–1.3	97	23/732	82/5000	56
α-MnO_2_-coated PANI/rGO	5–100	0.5 M H_2_SO_4_	−0.3-0.7	261	11/1250	75/2000	57
PANI@RGO.Ag	5–50	6 M KOH	0–1	385.4	27/1046.29	87.18/2000	58
Cu-PDA@rGO	10–50	1 M KOH	0–1.45	159.4	32.10/180.17	99.4/5000	This work

## 2 Experimental

### 2.1. Chemicals

Pyridine-3,5-dicarboxylic acid (CAS:499-81-0, purity ≥98%), dimethylformamide (CAS:62-12-2, purity ≥99.5%), copper acetate monohydrate (CAS:6046-91-1, purity ≥99%), activated carbon (CAS:4740-44-0), ammonium persulfate (CAS:7727-54-0, purity ≥98%), aniline (CAS: 65-53-3, purity ≥99.5%), *N*-methyl pyrrolidone (CAS:872-50-4, purity ≥99%), potassium hydroxide (CAS:1310-58-3, purity ≥90%), and hydrochloric acid (CAS:7647, purity ≥37%) were purchased and used without further purification.

### 2.2. Synthesis of Cu-PDA-MOF

**Fig. 1:**
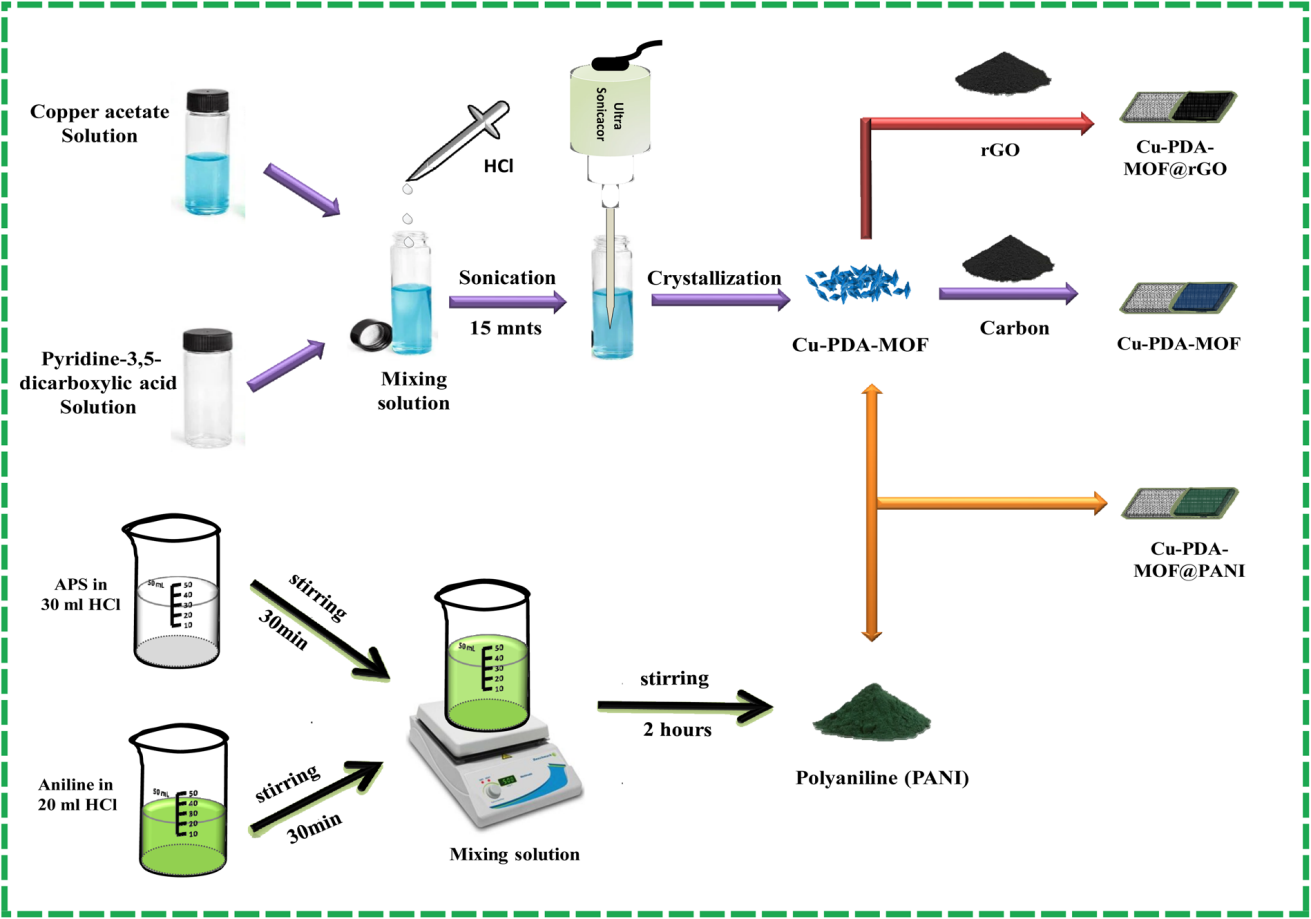
Schematic for the synthesis of the Cu-PDA-MOF, Cu-PDA@rGO and Cu-PDA@PANI electrodes.

Pyridine-3,5-dicarboxylic acid (41.7 mg, 0.25 mmol) was dissolved in a 5 mL mixture of DMF and H_2_O (30 : 70 v/v). Copper acetate monohydrate (49.91 mg, 0.25 mmol) was dissolved in 3 mL of the above mixture. The ligand and metal salt solutions were mixed with the addition of 3 drops of HCl and sonicated (MSE Sanyo, Soni Prep 150) for 15 minutes at an amplitude of 15 microns and a frequency of 23 kHz, followed by maintaining the temperature at 50 °C and providing slow evaporation. Dark blue needle-like crystals were obtained, washed with DMF and dried in the air (Fig. 1). Yield: *ca.* 59%.

### 2.3. Synthesis of polyaniline (PANI)

0.25 M aniline and 1 M aqueous HCl (20 mL) were mixed for about 30 minutes. 1 M HCl (30 mL) with 0.2 M APS (ammonium persulfate) was mixed separately and added slowly to an aniline solution. The green precipitates formed were washed with deionized water and acetone, yielding PANI in the emerald phase (Fig. 1).

### 2.4. Electrode fabrication of Cu-PDA, Cu-PDA@PANI and Cu-PDA@rGO

Nickel foam (NF) was selected as the conductive substrate for the deposition of materials to prepare working electrodes. Before use, NF was sonicated with HCl, ethanol and water to remove impurities and oxides. 8 mg active material was mixed with 1 mg each of activated carbon (AC) and polyvinylidene fluoride (PVDF) binder (80%:10%:10%) in a *N*-methyl pyrrolidone (NMP) solvent. The mixture was magnetically stirred for 4 hours to ensure a homogeneous slurry, which was deposited onto the NF using the drop casting method. The as-prepared electrode was dried at 70 °C for 5 hours. The same procedure was repeated to deposit Cu-PDA with rGO and PANI (1 : 1) to obtain Cu-PDA@rGO and Cu-PDA@PANI electrodes, respectively, for electrochemical analysis. An activated carbon electrode was also obtained similarly. The mass of NF was measured before and after deposition, which revealed that the active material loaded onto the working electrode was 4 mg. In order to improve the performance of the asymmetric device, eqn (1) was used to balance the mass and charge:^59^1
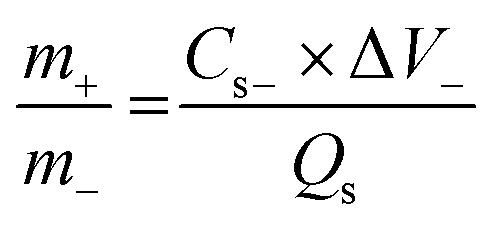
where “*m*,” “*C*_s_,” and “Δ*V*” represent the mass of the active material, specific capacities and operating voltages, with “+” and “–” indicating the positive and negative electrodes, respectively. The mass ratio was 1 : 2. The overall performance of the hybrid supercapacitor depends on the mass ratio of the negative and positive electrodes. Balanced charge storage ensures maximum specific power and specific energy, better cycling stability and an optimal potential window.

## 3 Characterization

### 3.1. Single crystal X-ray diffraction analysis of Cu-PDA MOF

**Fig. 2:**
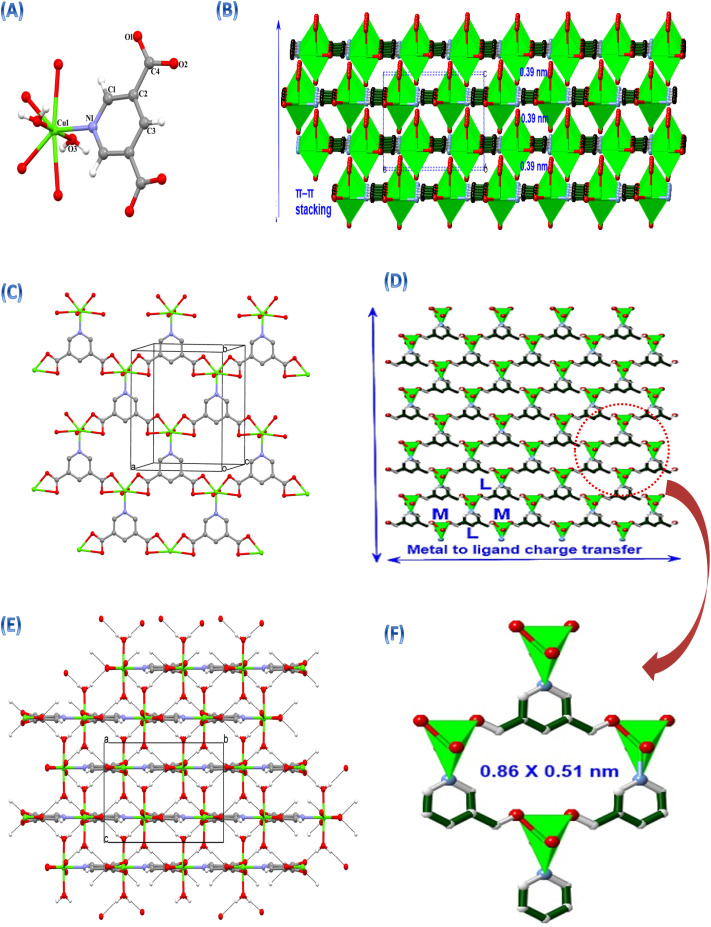
(A) Molecular structures of Cu-PDA showing an atom numbering scheme. (B) Stacking of a 2D MOF showing the π⋯π interactions. (C) Infinite ball and stick 2D supramolecular networks. (D and F) polyhedral schematic of metal-to-ligand charge transfer along the 2D and rectangular shaped pores. (E) Infinite 3D supramolecular network in Cu-PDA.

**Fig. 3:**
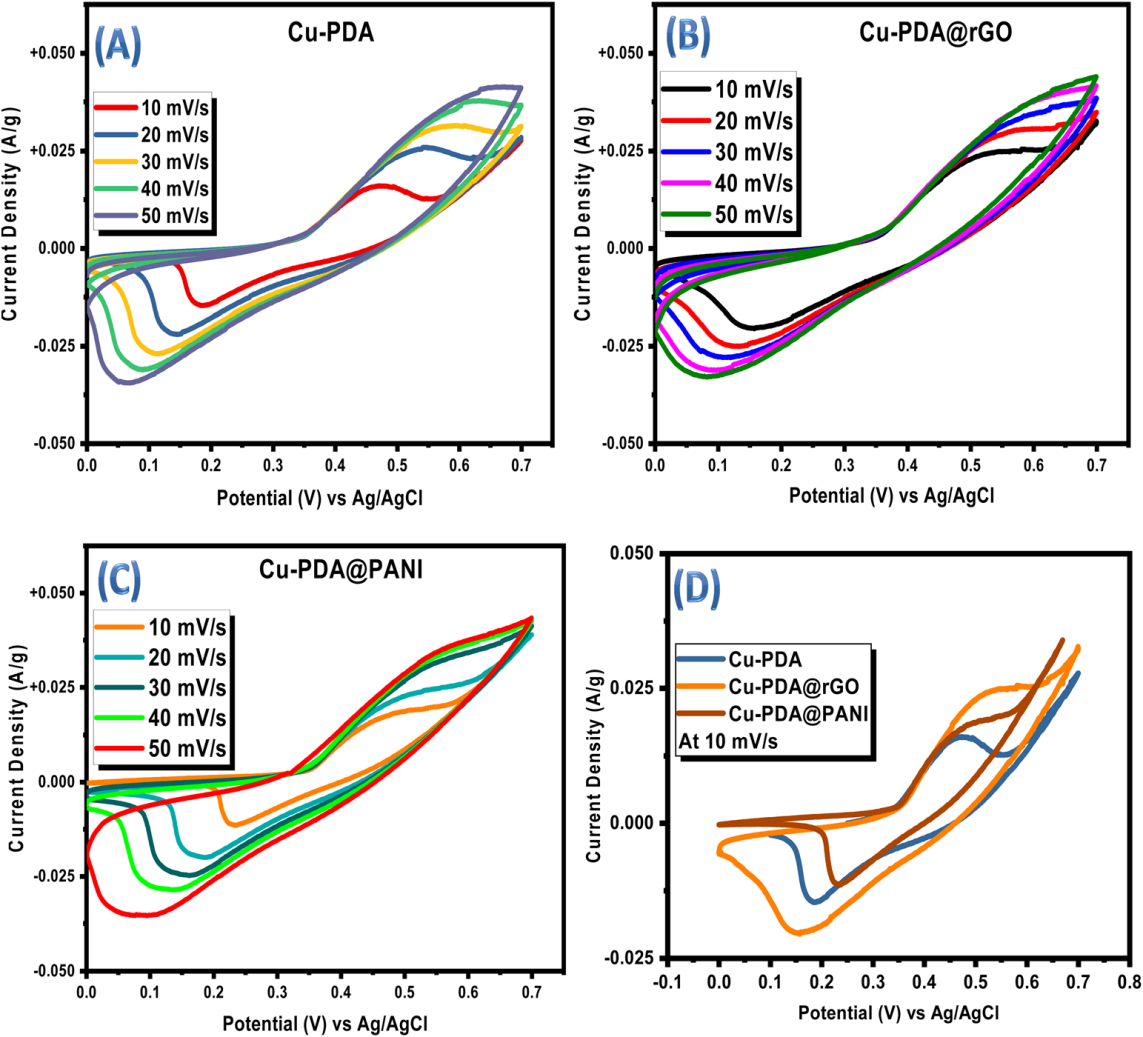
(A) CV plots of the Cu-PDA, (B) Cu-PDA@rGO, and (C) Cu-PDA@PANI electrodes. (D) CV comparison plot of the Cu-PDA, Cu-PDA@rGO and Cu-PDA@PANI electrodes at 10 mV s^−1^.

A suitable crystal of Cu-PDA was selected for data collection, which was performed using a Bruker diffractometer at 296 K. The H atoms of the aqua ligands were located on a difference map and refined freely. The other H atoms were located from difference maps and then treated as riding atoms with C–H distances of 0.93 Å. We used the following procedures for our analysis: solved using direct methods; refined by applying full-matrix least-squares methods (SHELXL-2013);^60^ molecular graphics: MERCURY;^61^ solution: WinGX; and data collection: Bruker APEX3.^62^ The molecular structure of Cu-PDA with the atom numbering scheme is shown in Fig. 2A. The molecular structure of complex Cu-PDA contains half Cu(ii) ion, half 3,5-pyridinedicarboxylic acid ligand and one aqua ligand. Crystal data and refinement parameters are illustrated in Table S1, while selected bond lengths and angles are presented in Table S2. The bond lengths and angles agree well with the literature values.^63^ The molecular structure of Cu-PDA with the atom numbering scheme is shown in Fig. 2A. The asymmetric unit of complex Cu-PDA contains a half Cu(ii) ion, a half 3,5-pyridinedicarboxylic acid ligand and one aqua ligand. The Cu(ii) ion is coordinated by four oxygen atoms and one nitrogen atom from 3,5-pyridinedicarboxylic acid ligands and two oxygen atoms from aqua ligands. Cu-PDA is a 2D coordination network (Fig. 2C and D) interconnected by utilizing a full coordination mode of 3,5-pyridinedicarboxylic acid, particularly through two carboxylate and one nitrogen. Molecules of Cu-PDA are linked by C–H⋯O and O–H⋯O hydrogen bonds (Table S3). Cu-PDA also contains π⋯π interactions. The perpendicular distance of pyridine rings is 3.327 Å, while the distance between the ring centroids is 3.914 Å (Fig. 2B). The slippage value is 1.586 Å. Stacking of 2D MOF is shown in Fig. 2B, while a schematic illustration of metal-to-ligand charge transfer along the *ab* plane in the 2D direction is presented in Fig. 2D, with a pore size of 0.86 × 0.51 nm shown in Fig. 3F. The aqua O3 atom acts as a hydrogen-bond donor to the O2 atom in the molecule at (–*x*, –*y* – 1, –*z*), thereby forming a centrosymmetric *R*_2_^2^(16) ring. Similarly, the aqua O3 atom acts as a hydrogen-bond donor to the O1 atom in the molecule at (*x* + 1/2, –*y* – 1/2, *z* + 1/2), thereby forming a centrosymmetric *R*_2_^2^(8) ring. The combination of these hydrogen bonds produces a 3D supramolecular network (Fig. 2E). A simulated XRD diffractogram is shown in Fig. S1. It is proposed that the improved electrochemical performance of 2D MOF may be attributed to charge transfer through-bond pathway, the extended conjugation pathway, through-space pathway, rectangular 1D pores and the nitrogen atom in the ligand.

### 3.2. PXRD analysis of PANI

The X-ray diffraction peaks of pure PANI (Fig. S2) showed an absence of sharp spectral peaks, confirming its amorphous nature. The average chain spacing of PANI was determined using eqn (2):^64^

2 *Nλ* = 2*d* sin *θ*

where *d* is the polymer inter chain spacing, *λ* represents the wavelength of the Cu, K radiation source, (*λ*) is 15 406 Å and *θ* is the diffraction angle between the scattering planes at the maximum intensity of the amorphous polymer and the incident ray. The average chain spacing (*S*) was estimated using Debye–Scherer's formula (eqn (3)):^65^

3 *D* = *Kλ*/*β* cos *θ*

where *D* is the crystallite size, *K* represents the shape factor, *θ* denotes the diffraction angle at a maximum peak intensity and *β* denotes the full width at half maximum *θ* in radian. The characteristic X-ray diffraction peak around 2*θ* = 25.89° was presented in the XRD, confirming the pattern of PANI. The presence of a broad band at an angle of 25.89° indicates periodic parallel and perpendicular polymeric chains of PANI. Table S4 lists the PXRD details of PANI.

### 3.3. Fourier transform infrared (FTIR) spectroscopy

In the FTIR spectrum (Fig. S3), the IR band of the pyridine-3,5-dicarboxylic acid showed two stretching modes at 3082 and 3375 cm^−1^: one can be attributed to alkene M(C–H), and the other S(O–H) stretching is generated by the carboxylic acid. The IR band at 3065 cm^−1^ indicates strong B(O–H) stretching, which can be assigned to the O–H of the carboxylic acid and free water molecules that take part in complexation. The presence of the S(C=O) stretching band of the carboxylic acid in the ligand is reduced in the complex. The band at 1600 cm^−1^ is possibly due to *ν*(COO^−^) stretching along with conjugated aromatic alkenes. The appearance of a strong stretching band at 1550 cm^−1^ indicates the involvement of the nitrogen atom of the ligand *ν*(C–N) in the coordination bond with the metal. The stretching modes observed at 1301 cm^−1^ for the ligand and 1378 cm^−1^ for the complex can be attributed to the S(C–N) stretching of the aromatic amine. The bands that appeared at 553–515 cm^−1^ were assigned to the M–O mode.^66^

In the FT-IR spectrum of pure PANI (Fig. S4), broad peaks in the 4000–3000 cm^−1^ range correspond to the *N*–H stretching vibration of the secondary amine. The peak at 1302 cm^−1^ is attributed to the C–N stretching of the primary aromatic amine, and the peak at 1150 cm^−1^ corresponds to the quinone ring of PANI.^67^ A band at 1649 cm^−1^ corresponds to the C–C stretching mode vibration, while the peaks at 1578 cm^−1^ and 1520 cm^−1^ are connected with C=N and C=C stretching on the aromatic ring, respectively.^68^

### 3.4. Thermogravimetric analysis (TGA)

The thermal decomposition of complex CP3 is illustrated in Fig. S5. At the first stage, two coordinated water molecules were released at about 170 °C, corresponding to a weight loss of 14.3% (calculated 13.6%). After the removal of the coordinated water molecules, the polymer was stable up to almost 290 °C. Beyond this point, at the second stage, the organic moiety was successively released up to 350 °C. Further increasing the temperature up to 650 °C leaves behind a residue of 42.3%, which may be attributed to a residue mixture of CuO and Cu_2_O (calcd, 42.2%).

### 3.5. Electrochemical calculations

The electrochemical performances of Cu-PDA, Cu-PDA@rGO and Cu-PDA@PANI were evaluated in a 1 M KOH aqueous electrolyte using CV, GCD and EIS. Half-cell analyses of all working electrodes were conducted using a three-electrode system, which identified the most effective material for the subsequent development of an asymmetric supercapacitor device in a two-electrode configuration. The power law is a theoretical method for differentiating between the battery and capacitive behaviors of electrodes by extracting the b-value. Eqn (4), derived from rearranging eqn (5), is employed to generate a linear plot, which enables the calculations of the variables ‘*a*’ and ‘*b*’ from the intercept and slope, respectively:

4 *i* = *av*^*b*^

5 log (*i*) = log (*a*) + *b* log (*v*)

Battery grade materials generally exhibit a *b*-values near 0.5, while capacitive materials tend to have values approaching 1.0. This criteria was utilized to evaluate the redox potential of the materials, and the specific capacitance was calculated at different scan rates using eqn (6):6
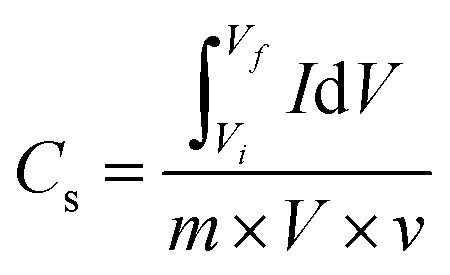


The numerator of this equation represents the polygon area under the curve, where *m* is the mass of the active materials, *V* denotes the potential window, and *v* represents the scan rate. GCD measurements were employed to measure the charging and discharging capabilities of the material, and the specific capacitance (F g^−1^) was calculated at each current density using eqn (7):7
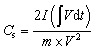


The integral in the numerator represents the integrated area under the discharging curve, where *I*/*m* is the current density and *V*^2^ is the square of the potential window. To calculate the specific capacity (C g^−1^) at each current density, eqn (8) was employed:8
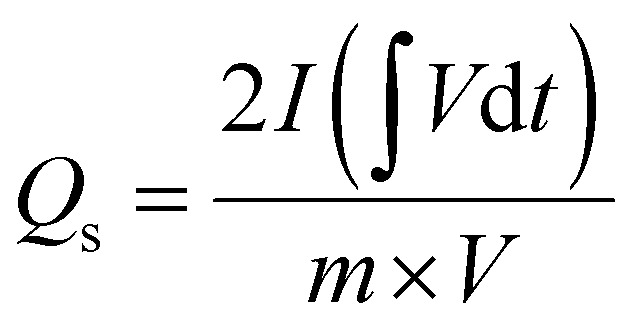


The primary distinction between the equations for calculating the specific capacitance and specific capacity lies in the denominators, where the former uses the square of the potential window and the latter utilizes the potential window. Eqn (9) and (10) were employed to calculate the specific energy *E*_s_ (Wh kg^−1^) and specific power *P*_s_ (W kg^−1^) of the materials, respectively:9
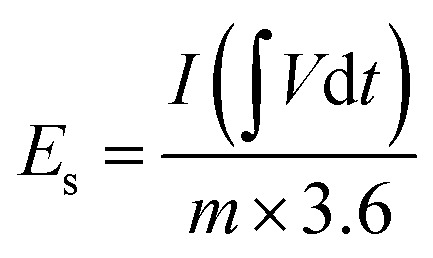
10
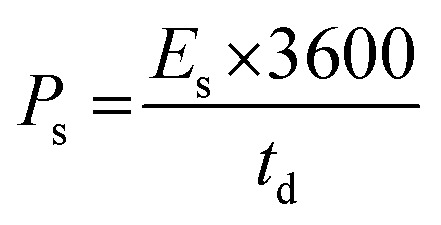
where *t*_d_ denotes the time taken for the electrode to discharge. A theoretical Dunn's method is employed to extract the diffusive and capacitive contributions at each scan rate. This method involves two equations, with eqn (11) representing the linear relationship used to obtain the *k*_1_ and *k*_2_ values from the resulting slope and intercept. These regression parameters were subsequently utilized to estimate the capacitive and diffusive contributions by rearranging eqn (11) to obtain eqn (12):^47^11
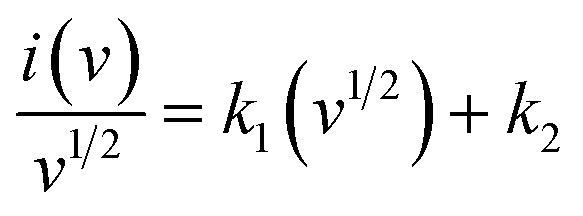
12
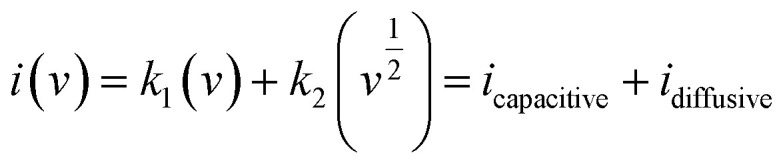


## 4 Results and discussion

### 4.1. Three-electrode (half-cell) electrochemical analyses of the Cu-PDA, Cu-PDA@rGO and Cu-PDA@PANI

Electrochemical analysis was performed on an Origalys electrochemical workstation using OrigaFlex-OGF05A and OrigaFlex-OGFEIS.

#### 4.1.1. Cyclic voltammetry

The redox reactions at the electrode–electrolyte interface were initially investigated using CV at the same set of scan rates in a potential window of 0 V to 0.7 V for Cu-PDA, Cu-PDA@rGO and Cu-PDA@PANI. CV provides insights into the reaction kinetics, electrochemical behavior and thermodynamics of the processes.^69^ The corresponding voltammograms for Cu-PDA, Cu-PDA@rGO and Cu-PDA@PANI are presented in Fig. 3A–C, respectively. These CV curves illustrate the relationship between the applied potential and the resulting current changes for each electrode material. The reactions at the electrode–electrolyte interface are initiated only upon the application of potential, which, when increased within the given potential window, energetically facilitates the reactions. This results in redox peaks for battery-grade materials and rectangular peaks for capacitive-grade materials. The redox activity in the CV curves is prominent for Cu-PDA and Cu-PDA@rGO, while Cu-PDA@PANI displays rectangular shapes in forward scan rates, with redox peaks in reverse scan rates. The battery-like characteristics of Cu-PDA and Cu-PDA**@rGO** are evident, although the negligible changes and uniformity in peak locations even at higher scan rates for Cu-PDA**@rGO** indicate its suitability as a pseudocapacitive material. Fig. 3D compares the CV profiles for Cu-PDA, Cu-PDA@rGO and Cu-PDA@PANI at a scan rate of 10 mV s^−1^. This comparison reveals that Cu-PDA@rGO has a larger area under the curve, which indicates its superior electrochemical potential compared to Cu-PDA and Cu-PDA@PANI.

**Fig. 4:**
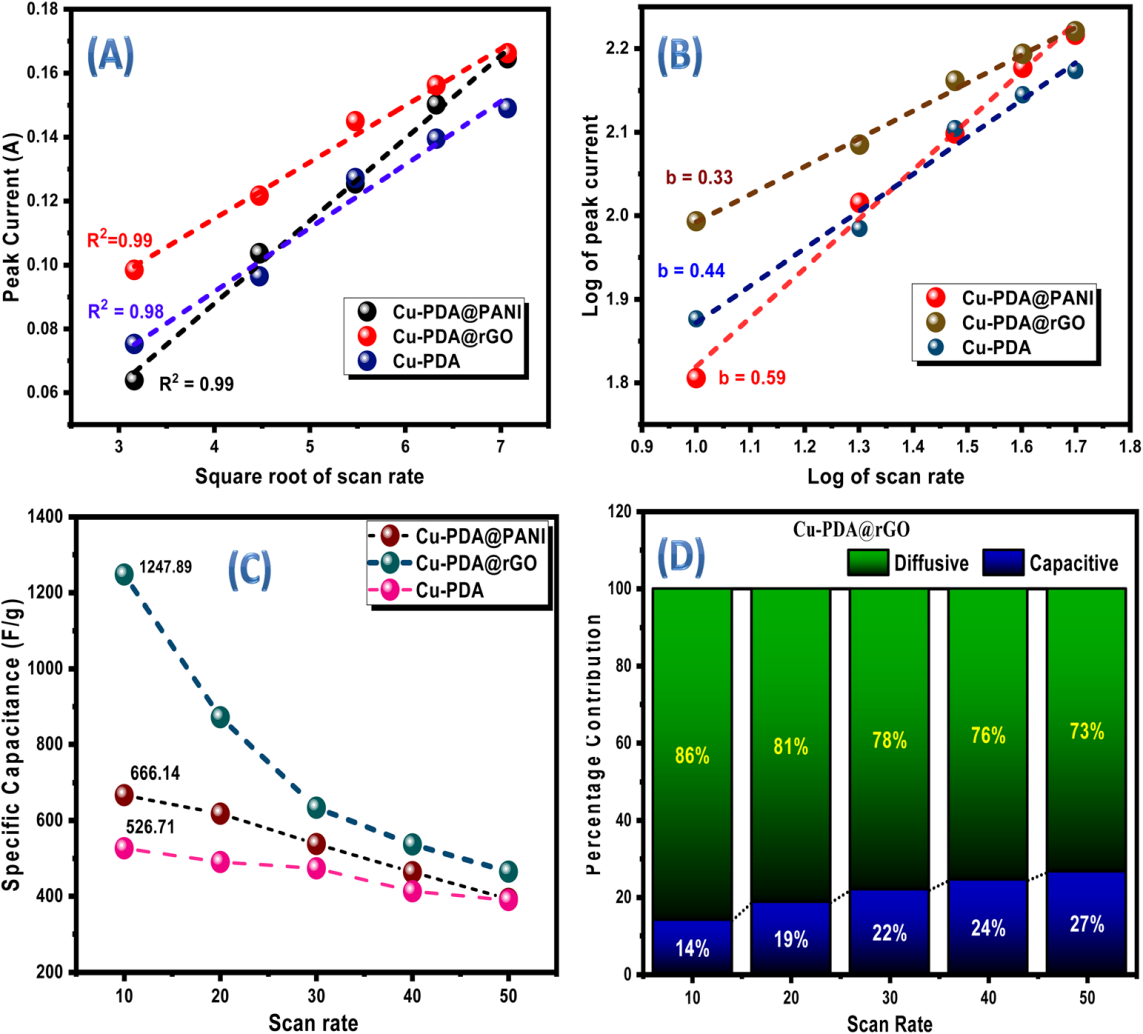
(A) Comparison of the linear regression fitting plot for the Cu-PDA, Cu-PDA@rGO, and Cu-PDA@PANI electrodes. (B) *b*-Value comparison plot for the Cu-PDA, Cu-PDA@rGO, and Cu-PDA@PANI electrodes. (C) Comparison of the specific capacitance at each scan rate for the Cu-PDA, Cu-PDA@rGO and Cu-PDA@PANI electrodes. (D) Percentage contribution bar graph at each scan rate for the Cu-PDA@rGO electrode.

To further elucidate the electrochemical properties, *R*^2^ values were compared by plotting the peak current against the square root of the scan rate, as shown in Fig. 4A. A linear relationship between peak currents and the square root of the scan rate indicates reversibility. Cu-PDA@PANI and Cu-PDA@rGO exhibited *R*^2^ values of 0.99. In contrast, Cu-PDA with an *R*^2^ value of 0.98 exhibited a slight deviation. The power law, articulated in eqn (4) and (5), was employed to establish a linear fit between the logarithm of the scan rate and the logarithm of the peak current, as depicted in Fig. 4B. The *b*-values for Cu-PDA, Cu-PDA@rGO and Cu-PDA@PANI were determined to be 0.59, 0.33 and 0.44, respectively. These values suggest that the material possesses a battery-like nature. Furthermore, specific capacitance was calculated using eqn (6), as shown in Fig. 4C. The highest specific capacitance was recorded for Cu-PDA@rGO with a value of 1247.89 F g^−1^, followed by 526.71 F g^−1^ for Cu-PDA and 666.14 F g^−1^ for Cu-PDA@PANI. These electrochemical outcomes highlighted the promising potential of Cu-PDA**@rGO** as a charge storage material. Based on its exceptional performance, Cu-PDA@rGO was selected to employ Dunn's method using eqn (11) and (12). The percentage contribution for Cu-PDA@rGO in a three-electrode system at each scan rate is depicted in Fig. 4D. It is observed that the diffusive contribution was predominant at lower scan rates but decreased with increasing scan rates. This trend can be attributed to the emergence of EDLC at elevated scan rates, which enhanced the overall capacitive response.

#### 4.1.2. Galvanostatic charge–discharge (GCD)

**Fig. 5:**
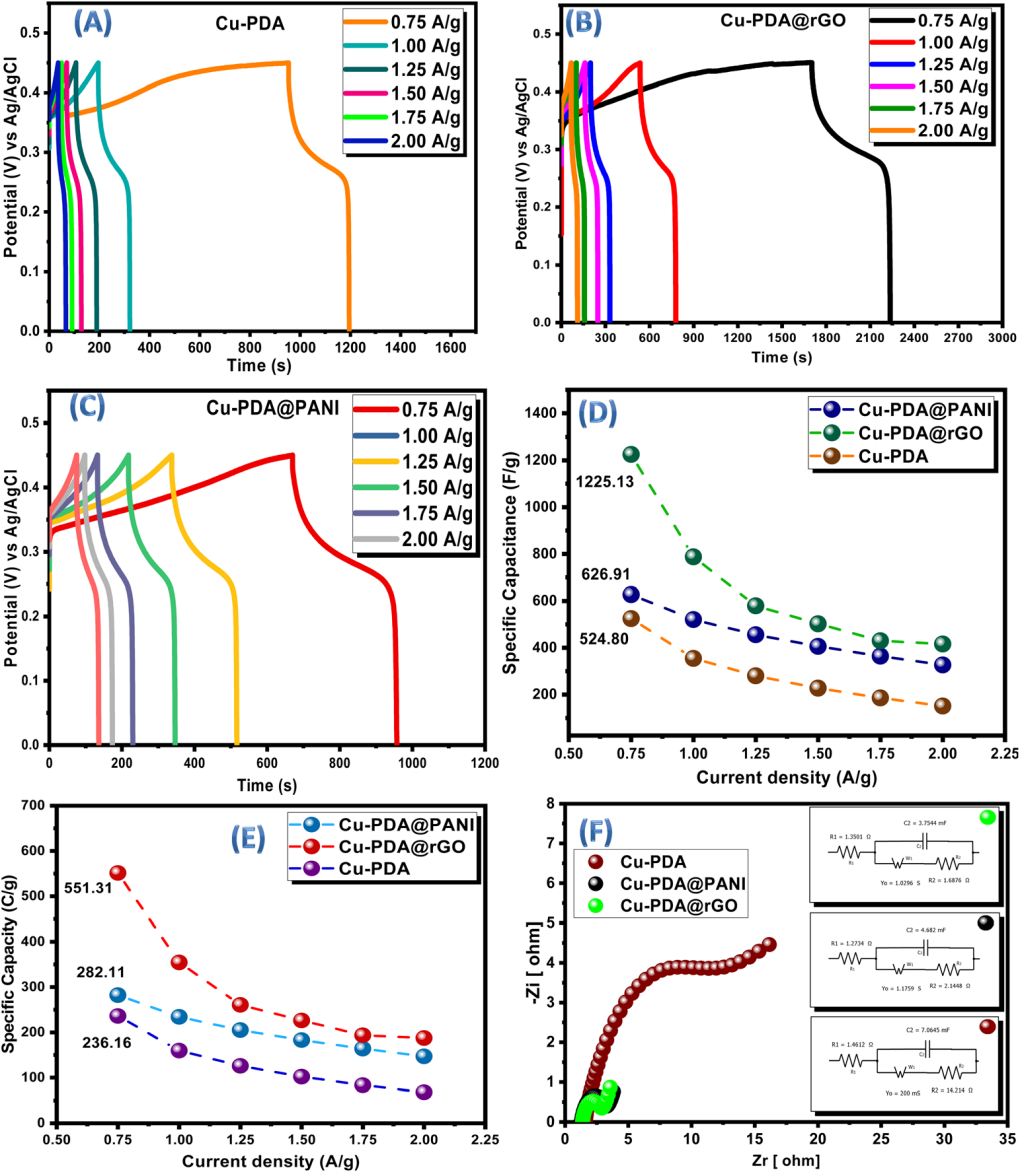
(A) GCD plots of Cu-PDA, (B) GCD plots of Cu-PDA@rGO and (C) GCD plots of Cu-PDA@PANI. (D) Comparison of the specific capacitance at each current density for the Cu-PDA, Cu-PDA@rGO and Cu-PDA@PANI electrodes and (E) comparison of the specific capacity at each current density for the Cu-PDA, Cu-PDA@rGO and Cu-PDA@PANI electrodes. (F) Nyquist plots with circuit fitting for the Cu-PDA, Cu-PDA@rGO and Cu-PDA@PANI electrodes.

GCD tests were executed at different current densities (0.75 to 2 A g^−1^) to evaluate the charging and discharging capabilities of the materials at a potential window ranging from 0 V to 0.45 V. These GCD results not only provided insights into the electrochemical performance but also served to corroborate the reliability and accuracy of the CV analysis. The GCD profiles for Cu-PDA, Cu-PDA@rGO and Cu-PDA@PANI conducted at comparable current densities are illustrated in Fig. 5A–C, respectively. Cu-PDA and Cu-PDA**@rGO** displayed more pronounced humps and greater symmetry in their charging and discharging curves than Cu-PDA@PANI. In a supercapacitor, charging time depends on applied voltage, capacitance and electrical resistance. A high capacitance of the supercapacitor and a low current are required to prevent damage, which takes a considerable time for charging. Moreover, during electric double layer (EDL) formation, physical adsorption of electrolyte ions at the electrode surface occurs quickly due to electrostatic attraction. On the other hand, during discharging, the desorption of ions is more rapid due to electrostatic repulsion between the electrode surface and ions. It is also considerable that charging and discharging times also depend on the nature of the electrode material. The specific capacitance for each electrode material was determined at each current density using eqn (7), with the results depicted in Fig. 5D. This trend is consistent with the specific capacitance derived from the CV data. Moreover, specific capacities were measured at each current density using eqn (8). The results are illustrated in Fig. 5E. Cu-PDA@rGO exhibited the highest specific capacity with 551.31 C g^−1^, followed by 236.16 C g^−1^ for Cu-PDA and 282.11 C g^−1^ for Cu-PDA@PANI. The rGO is a flat, sheet-like 2D structure that provides excellent conductivity^70^ and a large surface area for adsorption and reaction processes,^71^ so it provides the highest specific capacitance in comparison to the Cu-PDA or Cu-PDA@PANI electrode individually.

#### 4.1.3. Electrochemical impedance spectroscopy

A pivotal technique for characterizing any energy storage system in terms of the total conductivity is facilitated through EIS, which is conducted at an AC frequency ranging from 0.1 Hz to 100 KHz. Fig. 5F presents the comparative Nyquist circuit fitted plot for these materials. The total conductivity of the system is typically defined using three key parameters: equivalent series resistance (ESR), charge transfer resistance (*R*_ct_) and Warburg impedance. ESR is extracted from the *x*-axis intercept in the high frequency region. The ESR values were found to be 1.46 Ω for Cu-PDA, 1.35 Ω for Cu-PDA@rGO and 1.27 Ω for Cu-PDA@PANI. A higher ESR indicates increased internal resistance and lower efficiency within a system. *R*_ct_ is the resistance encountered by ions as they diffuse into the electrodes for electrochemical reactions. *R*_ct_ is manifested by semicircles in the high frequency region. Cu-PDA@rGO exhibited a lower *R*_ct_ of 1.69 Ω compared to 14.21 Ω for Cu-PDA and 2.14 Ω for Cu-PDA@PANI. An elevated *R*_ct_ implies a more substantial barrier at the electrode–electrolyte interface, which impedes the movement of charges or ions. Moreover, the Nyquist plots do not exhibit significant Warburg impedance, which can be indicated by the extended line in the low frequency region. The capacitance values measured at the electrode–electrolyte interface revealed that of Cu-PDA@rGO (3.75 mF) to be lower than those of Cu-PDA (7.05 mF) and Cu-PDA@PANI (4.68 mF). These findings suggest that the Cu-PDA@rGO system is predominantly controlled by faradaic processes, while non-faradaic processes are promoted in Cu-PDA and Cu-PDA@PANI systems.

### 4.2. Asymmetric supercapacitor assembly (Cu-PDA@rGO//AC)

**Fig. 6:**
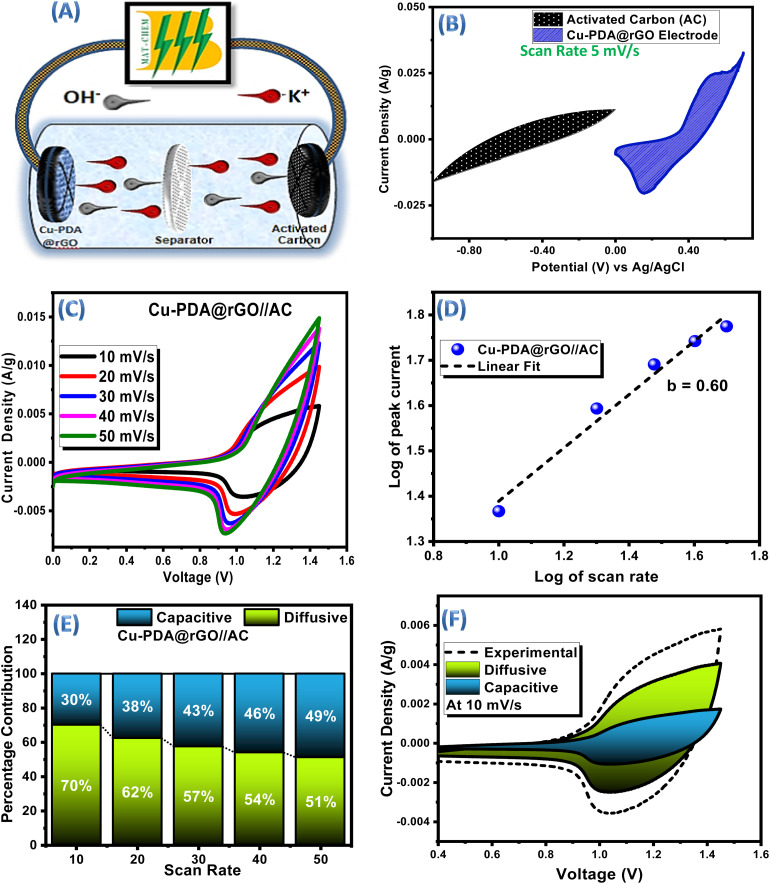
(A) Hypothetical diagram of the asymmetric supercapacitor device. (B) CV comparison plot of the Cu-PDA@rGO and AC electrodes at 5 mV s^−1^ (C) CV plots of the Cu-PDA@rGO//AC device. (D) *b*-Value of Cu-PDA@rGO//AC. (E) Percentage contribution bar chart at each scan rate for the Cu-PDA@rGO//AC device. (F) Pictorial contribution of the diffusive and capacitive elements in an asymmetric device at 10 mV s^−1^.

Among the materials characterized, Cu-PDA@rGO emerged as a standout material, and it was subsequently chosen as the positive electrode against activated carbon behaving as the negative electrode for making an asymmetric supercapacitor, *i.e.*Cu-PDA@rGO//AC (Fig. 6A). Independent CVs of Cu-PDA@rGO and AC electrodes are shown in Fig. 6B, which helps determine a potential window of 0 V to 1.45 V for CV analysis. The CV profile of activated carbon exhibits a rectangular shape, while that of Cu-PDA@rGO displays distinct redox peaks. CV was performed for the hybrid device at different scan rates (Fig. 6C). Linear fitting of the CV data achieved an *R*^2^ value of 0.98, which demonstrated excellent reversibility and a *b*-value of 0.60 (Fig. 6D), further substantiating the presence of both faradaic and non-faradaic processes occurring in the device. Employing Dunn's method in the light of eqn (11) and (12), the percentage contributions of capacitive and diffusive elements at varying scan rates were quantified (Fig. 6E). At a scan rate of 10 mV s^−1^, the diffusive contribution was 70%, and 30% part was capacitive. At a scan rate of 50 mV s^−1^, the diffusive contribution was 51%, and the capacitive part was equivalent to 49%. This shift suggests that enhanced capacitive contributions, rising from 30% to 49%, are attributable to the development of EDLC. Consequently, it can be inferred that at a scan rate of 50 mV s^−1^, faradaic and non-faradaic reactions coexist equally. The capacitive and diffusive contributions at a 10 mV s^−1^ scan rate are shown in Fig. 6F for clarification.

**Fig. 7:**
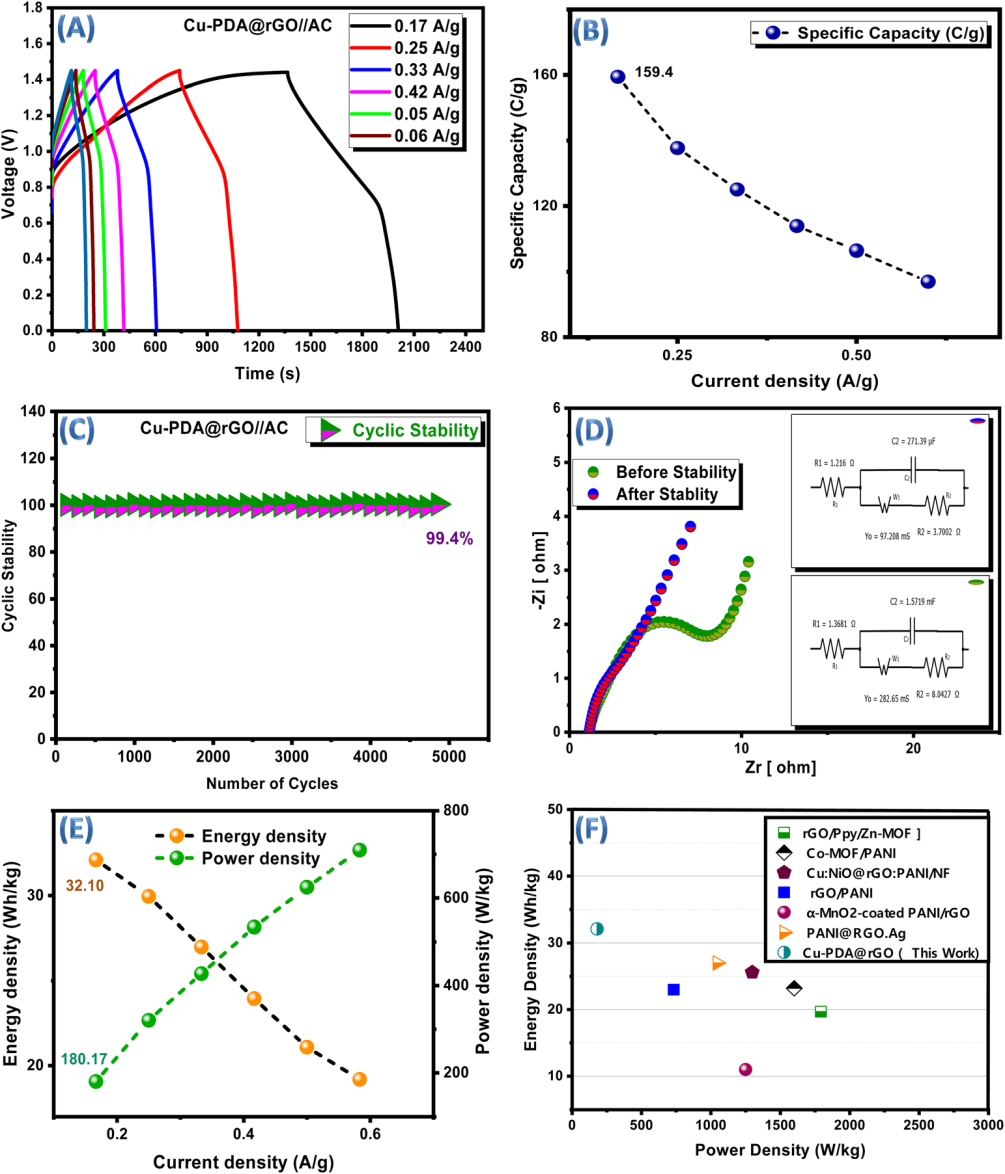
(A) GCD plots of Cu-PDA@rGO//AC. (B) Comparison of the specific capacity at each current density in Cu-PDA@rGO//AC. (C) Coulombic efficiency of Cu-PDA@rGO//AC. (D) EIS Nyquist plots with circuit-fitted diagrams (before and after stability tests) for Cu-PDA@rGO//AC. (E) Comparison of the specific energy and specific power with the current density for Cu-PDA@rGO//AC. (F) Ragone plot.

GCD curves for the asymmetric device at different current densities (Fig. 7A) exhibited both linear and non-linear segments. The linear segments reflected their capacitive behavior, while the non-linear segments with subtle humps indicated the simultaneous presence of both faradaic and non-faradaic reactions. The GCD data were subsequently used to retrieve specific capacity at each current density for the asymmetric device. Its trend is shown in Fig. 7B. The highest specific capacity achieved was 159.4 C g^−1^ at 0.16 A g^−1^. The shelf life for performance of the device was evaluated, which demonstrated an impressive 99.4% coulombic efficiency potential maintained after 5000 GCD cycles, as shown in Fig. 7C. A slight decrease in coulombic efficiency was observed when the number of GCD cycles increased. For calculating key device metrics, eqn (9) and eqn (10) were utilized to determine energy and power densities at each current density. Their correlation graph is shown in Fig. 7E. The device achieved a specific energy of 32.10 Wh kg^−1^ and a specific power of 180.17 W kg^−1^.

By employing electronic impedance spectroscopy, it was demonstrated that in the three-electrode system, Cu-PDA@rGO exhibited an ESR of 1.35 Ω, *R*_ct_ of 1.69 Ω and a capacitance of 3.75 mF. The overall conductivity in the two-electrode system was also assessed both before and after stability tests. The Nyquist circuit fitted plots are shown in Fig. 7D. An ESR shift from 1.37 Ω to 1.22 Ω, capacitance shift from 1.57 mF to 27.39 µF, and an *R*_ct_ shift from 8.04 Ω to 3.76 Ω after stability tests indicated a less resistive nature and improved electrochemical conductivity. Moreover, a smaller semicircle diameter at the high frequency region and a larger slope indicate the faster diffusion of ions throughout the electrolyte, resulting in a higher capacitance of the hybrid device.^72^ Equivalent circuit fitting parameters are shown in Table 2.

**Table 2:** Equivalent circuit fitting parameters

Material	Equivalent series resistance (ESR) (Ω)	Warburg impedance (*W*) (S)	Charge transfer resistance (*R*_ct_) (Ω)	Capacitance (*C*_dl_) (mF)
Cu-PDA	1.46	0.20	14.21	7.05
Cu-PDA@rGO	1.35	1.18	1.69	3.75
Cu-PDA@PANI	1.27	1.03	2.14	4.68
Cu-PDA@rGO//AC (before stability)	1.37	0.28	8.04	1.57
Cu-PDA@rGO//AC (after stability)	1.21	0.97	3.70	0.27

## 5 Conclusions

Cu-PDA-MOF was synthesized from the heteroatomic linker pyridine-3,5-dicarboxylic acid and incorporated with reduced graphene oxide and polyaniline to improve its electrochemical properties. A 2D-layered structure and the presence of a nitrogen atom improve conductively but amalgamating conductive polymers, like reduced graphene oxide (rGO) and polyaniline (PANI), show excellent electrochemical performance. A three-electrode assembly helped to differentiate electrochemical performance, and Cu-PDA@rGO was found to be the most efficient electrode material. Hence, it was fabricated as a cathode in an asymmetric device against an activated carbon anode. The hybrid device exhibited specific capacity, specific energy and specific power of 159.4 C g^−1^, 32.10 Wh kg^−1^, and 180.17 W kg^−1^, respectively. After running 5000 GCD cycles, the device showed 99.4% coulombic efficiency. These detailed electrochemical outcomes of the synthesized materials demonstrate that this study is a positive addition to the current sustainable research in energy storage technology.

## Author contributions

Shahbaz: writing draft, Madiha: validation, Ghulam Ullah: characterization, Waheed: methodology, Maham: investigation, Sundas: formal analysis, Ayesha: data curation, Zeeshan: review, Onur: XRD software, and Shahzad: supervision.

## Conflicts of interest

The authors declared that they have no competing interests.

## Abbreviations

PDA: pyridine-3,5-dicarboxylic acid
SXRD: Single crystal X-ray diffraction
NF: Nickel foam
PVDF: Polyvinylidene fluoride
NMP: *N*-methyl pyrrolidone
CV: Cyclic voltammetry
GCD: Galvanostatic charge–discharge
EIS: Electronic impedance spectroscopy
EDLC: Electric double layer capacitor
*C*_s_
: Specific capacitance
*Q*_s_
: Specific capacity
ESR: Equivalent series resistance
*R*_ct_
: Charge transfer resistance
*E*_s_
: Specific energy
*P*_s_
: Specific power

## Supplementary Material

RA-016-D5RA05415J-s001

RA-016-D5RA05415J-s002

## Data Availability

CCDC 2389095 contains the supplementary crystallographic data for this paper.^73^ The data can be made available upon request for the manuscript. Supplementary information (SI) is available. See DOI: https://doi.org/10.1039/d5ra05415j.
