# Supplementary material for: Tuning the electrochemical performance of a copper-based 2D rectangular layered metal organic framework by incorporating reduced graphene oxide and polyaniline
Source: RSC Adv. 2026 Mar 17;16(17):15036–50. doi: 10.1039/d5ra05415j (PMC12994482; doi:10.1039/d5ra05415j)
Supplement: RA-016-D5RA05415J-s001 [file RA-016-D5RA05415J-s001.pdf]

Supplementary Information (SI) for RSC Advances.
This journal is © The Royal Society of Chemistry 2026

Tuning the electrochemical performance of copper-based 2D rectangular layered Metal Organic Framework by incorporating reduced-graphene oxide and polyaniline

*Muhammad Shahbaz[a], Madiha Riasat[a], Ghulam Ullah[a], Muhammad Waheed Mushtaq[b], Maham Saeed[a], Sundas Shahzad[a] , Ayesha Shahzad, Zeeshan Mustafa[c], Onur Şahin[d], Shahzad Sharif[a]**

Supplementary Information

Table 1: Crystal data and structure refinement parameters for **Cu-PDA MOF.**

Empirical formula	$C_7H_7CuNO_6$
Formula weight	264.68
Crystal system	Monoclinic
Space group	*C2/c*
a (Å)	10.101(3)
b (Å)	11.843(3)
c (Å)	7.0691(19)
β (°)	105.308(6)
V (Å^3)	815.6(4)
Z	4
D_c (g cm^{-3})	2.155
μ (mm^{-1})	2.69
θ range (°)	3.1-28.2
Measured refls.	9406
Independent refls.	758
R_{int}	0.034
S	1.10
R1/wR2	0.041/0.116
$\Delta\rho_{max}/\Delta\rho_{min}$ (eÅ^{-3})	1.95/-0.56
CCDC	2389095

Table 2: Selected bond distances and angles(Å, º)

Cu1-N1	2.102(4)	Cu1-O1^{i}	2.188(2)	Cu1-O3	1.932(3)
C3-C2	1.386(4)	C4-O2	1.242(4)	C1-N1	1.345(4)
C1-N1-Cu1	121.21(19)	O3-Cu1-N1	88.64(7)	O3-Cu1-O1^{ii}	94.49(10)
N1-Cu1-O1^{i}	137.67(7)	O1-C4-C2	119.5(3)	O2-C4-O1	122.9(3)

Symmetrycodes: (i) −*x*−1/2, *y*+1/2, −*z*−1/2; (ii) *x*+1/2, *y*+1/2, *z*.

Table 3: Hydrogen bond parameters (Å, °)

D-H···A	D-H	H···A	D···A	D-H···A
C1—H1···O2^{iv}	0.93	2.26	2.981 (5)	134
O3—H3A···O2^{v}	0.80 (2)	1.93 (2)	2.717 (4)	169
O3—H3B···O1^{vi}	0.81 (2)	1.95 (2)	2.755 (3)	173

Symmetrycodes: (iv) −x−1/2, y+1/2, −z−1/2; (v) −x, −y−1, −z; (vi) x+1/2, −y−1/2, z+1/2.

Table.4: PXRD information ofPANI

**Samples**	**2θ(deg.)**	**θ(deg.)**	**d (nm)**	**FWHM (β)**	**D(nm)**
Polyaniline	25.89°	12.94°	0.3622	0.269	63

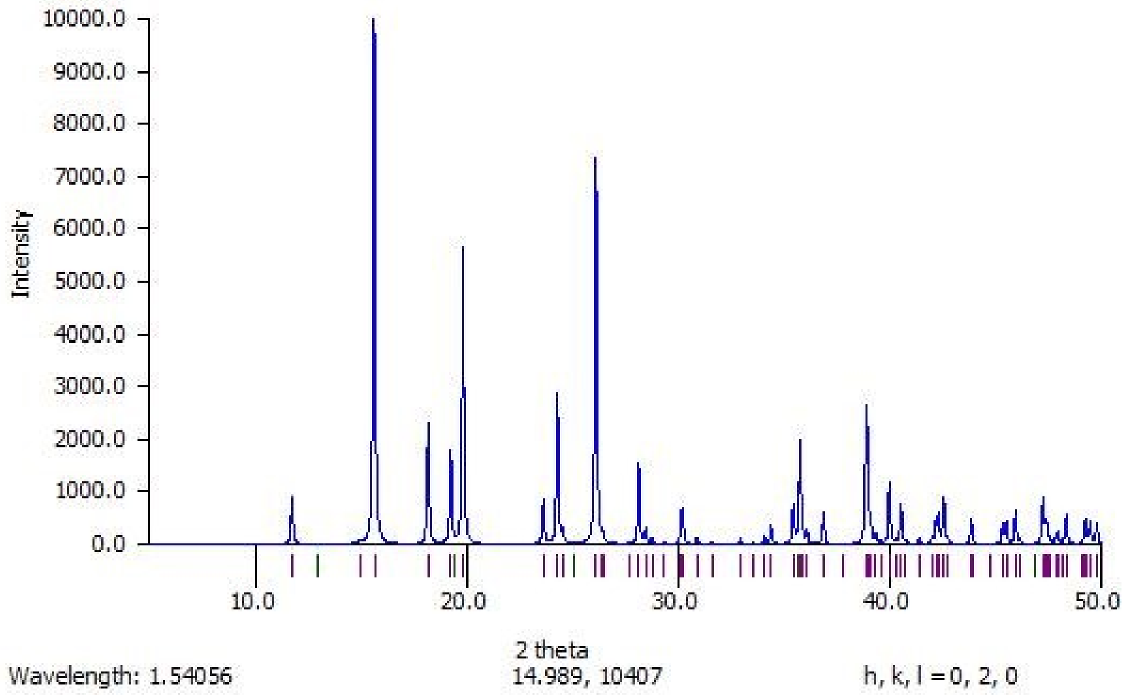

Fig. S1. Simulated XRD diffractogram of Cu-PDA-MOF

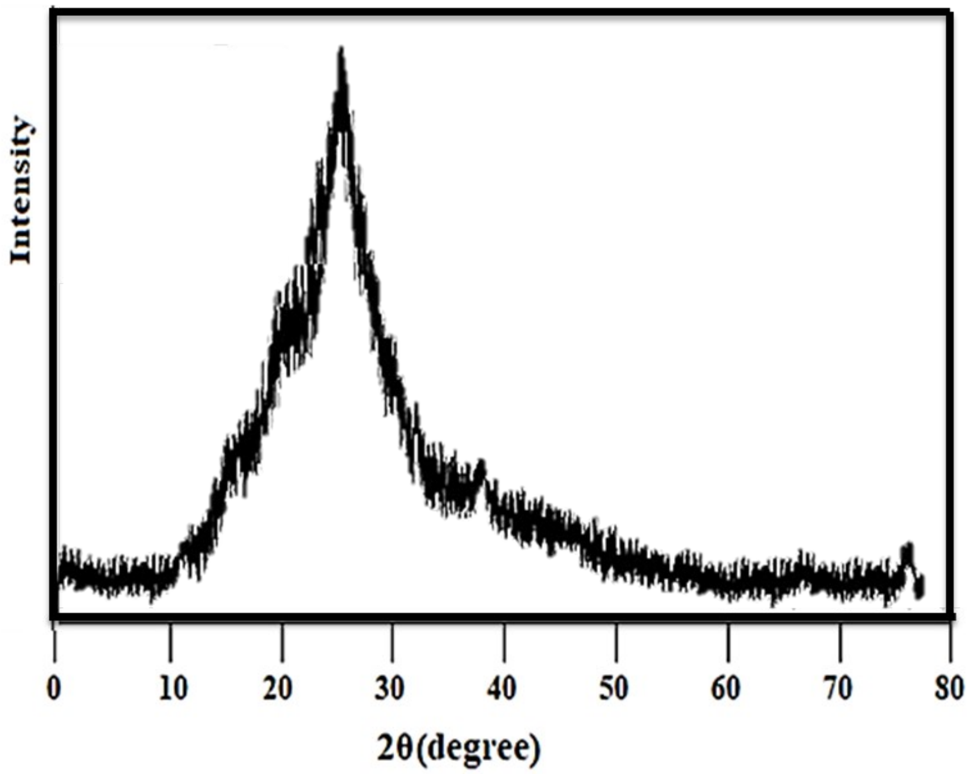

Fig. S2: PXRD spectra of PANI

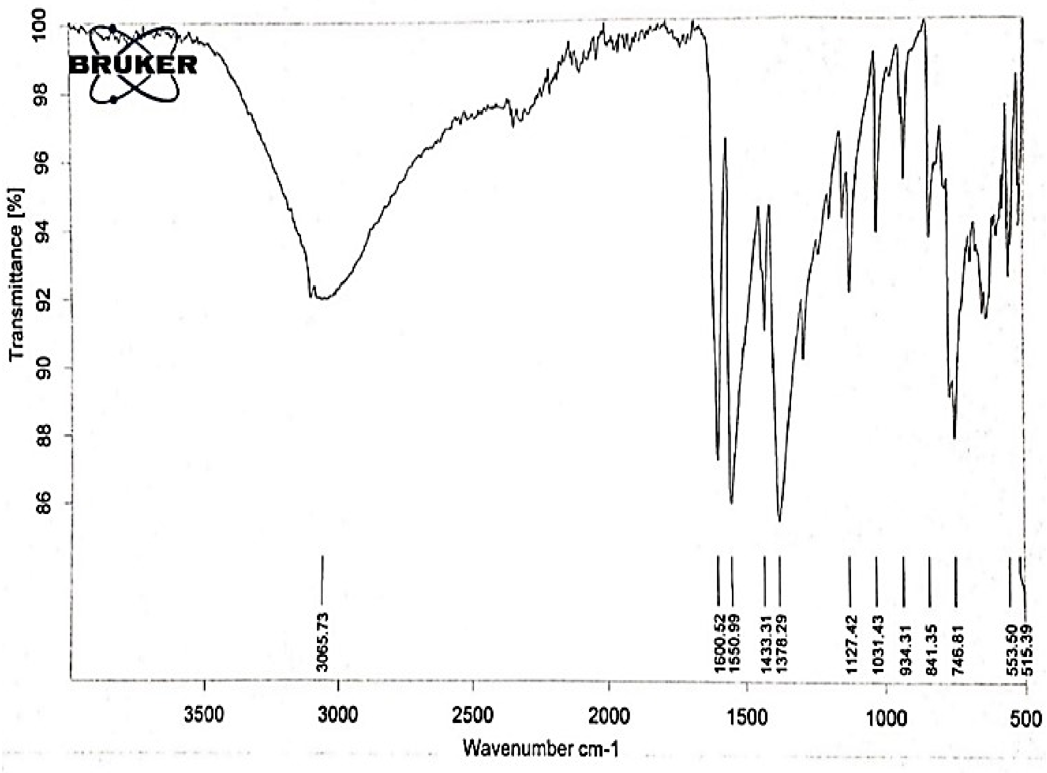

Fig.S3: FTIR spectra of Cu-PDAMOF

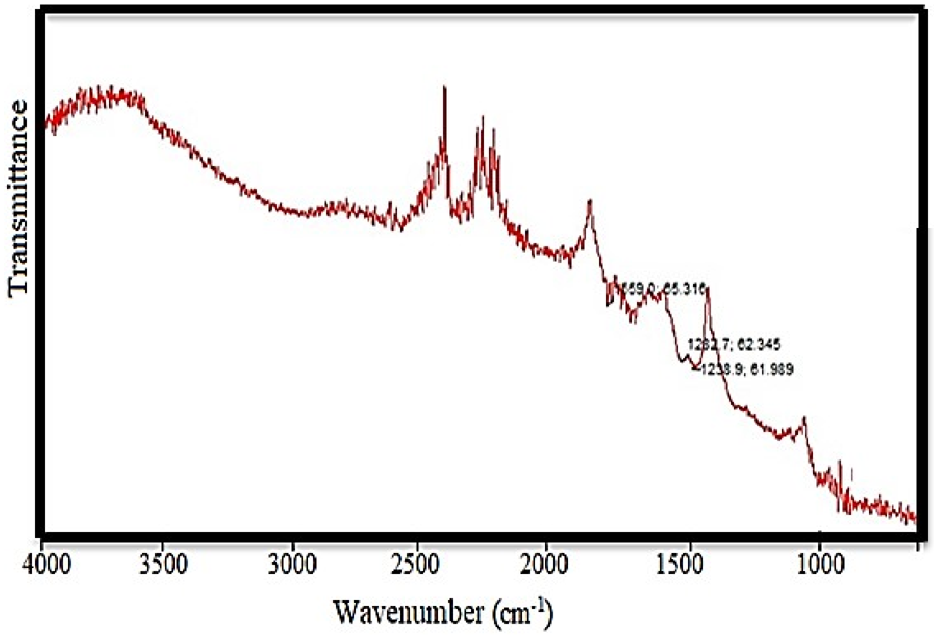

Fig. S4: FTIR spectra of PANI

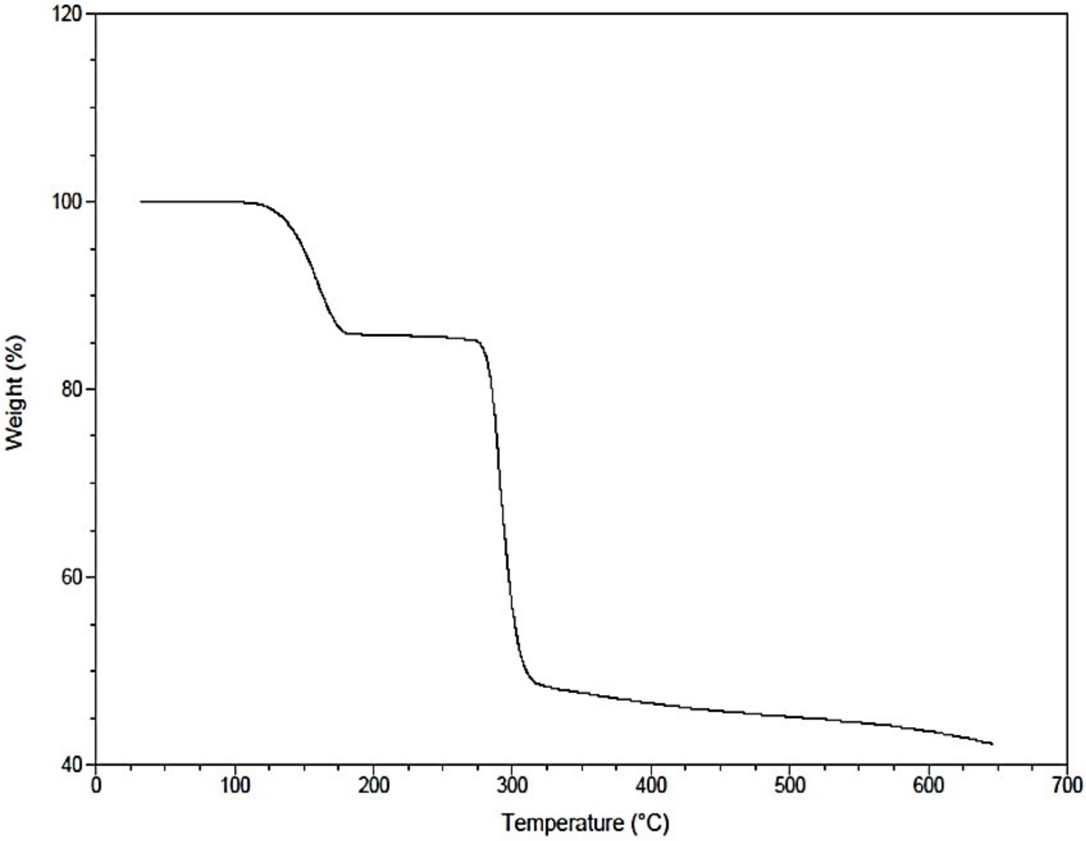

Fig.S5: TGA of Cu-PDAMOF
